# Buyang Huanwu Decoction Alleviates Chronic Intermittent Hypoxia–Induced Myocardial Inflammation and Fibrosis via the NF‐*κ*B/LOX Signaling Pathway

**DOI:** 10.1155/humu/2462859

**Published:** 2026-02-05

**Authors:** Lijun Ou, Chenyi Liu, Weicheng Zhao, Cairu Liu, Ye Ning

**Affiliations:** ^1^ Department of Cardiovascular Disease, The Fourth Clinical College of Guangzhou University of Traditional Chinese Medicine/Shenzhen Traditional Chinese Medicine Hospital, Shenzhen, China; ^2^ Emergency Department, Shenzhen Luohu Hospital of Traditional Chinese Medicine, Shenzhen, China

**Keywords:** Buyang Huanwu decoction (BYHW), cardiac function, chronic intermittent hypoxia (CIH), Lysyl oxidase (LOX), myocardial fibrosis, nuclear factor-kappa B (NF-)

## Abstract

**Objective:**

To investigate the protective effects of Buyang Huanwu decoction (BYHW) on myocardial injury induced by chronic intermittent hypoxia (CIH) in rats and to analyze its potential regulatory mechanism through the NF‐*κ*B/LOX signaling pathway.

**Methods:**

Then, 36 adult male Sprague–Dawley (SD) rats (weighing 200–250 g) were randomly divided into six groups (*n* = 6): normal control (NC), CIH model, BYHW, BYHW + lipopolysaccharide (LPS), BYHW + pyrrolidinedithiocarbamate (PDTC), and LPS. Except for the NC group, all groups underwent 5 weeks of intermittent hypoxia (8 h/day) alongside their respective drug treatments. Postintervention, systolic blood pressure and heart rate were recorded. Cardiac function was evaluated by echocardiography to measure left ventricular ejection fraction (LVEF), left ventricular fractional shortening (LVFS), and left ventricular internal dimension at end‐diastole (LVDd) and left ventricular internal dimension at end‐systole (LVDs). Myocardial sections were analyzed by HE and Sirius Red staining for quantitative assessment of inflammatory cell infiltration and collagen volume fraction (CVF). The protein and mRNA expression levels of NF‐*κ*B, LOX, Collagen I, and Collagen III in cardiac tissue were analyzed by Western blot and qPCR.

**Results:**

Compared with the NC group, rats exposed to CIH or LPS showed elevated blood pressure and an increased heart rate, along with impaired cardiac function, as evidenced by reduced LVEF (*p* < 0.05) and LVFS (*p* < 0.05), along with increased LVDd (*p* < 0.05) and LVDs (*p* < 0.05). BYHW treatment significantly ameliorated the CIH‐induced cardiac dysfunction and ventricular dilation (*p* < 0.05), and these improvements were further enhanced by cotreatment with BYHW and the NF‐*κ*B inhibitor PDTC (*p* < 0.05). Conversely, cotreatment with BYHW and the NF‐*κ*B agonist LPS attenuated the cardioprotective effects of BYHW (*p* < 0.05). Histologically, BYHW significantly reduced CIH‐induced myocardial structural disruption, inflammatory cell infiltration, and fibrosis (CVF) (*p* < 0.05), effects that were potentiated by cotreatment with BYHW and PDTC, and counteracted by cotreatment with BYHW and LPS. At the molecular level, Western blot and qPCR analyses revealed that BYHW significantly reduced both the protein and mRNA levels of NF‐*κ*B, LOX, Collagen I, and Collagen III (*p* < 0.05) compared with the NC group. This suppression was further enhanced by cotreatment with BYHW and PDTC and attenuated by cotreatment with BYHW and LPS.

**Conclusion:**

BYHW improves cardiac function and reduces myocardial inflammation and fibrosis in SD rats with CIH. This effect may be related to the inhibition of the NF‐*κ*B/LOX signaling pathway. Cotreatment with BYHW and PDTC enhances this therapeutic effect, while cotreatment with BYHW and LPS attenuates this effect. This study provides preliminary experimental evidence for the cardioprotective effects of BYHW.

## 1. Introduction

Obstructive sleep apnea (OSA) is a common sleep‐related breathing disorder characterized by recurrent upper airway obstruction and chronic intermittent hypoxia (CIH) during sleep [1]. A growing body of epidemiological evidence indicates that OSA is closely associated with various cardiovascular diseases, including hypertension, arrhythmia, myocardial infarction, and heart failure, and has been identified as an independent risk factor for sudden cardiac death [1, 2]. Among the multiple pathophysiological mechanisms, CIH is recognized as a central contributor to cardiovascular injury in OSA, promoting myocardial structural and functional remodeling through oxidative stress, inflammation, autonomic imbalance, and other signaling pathways [3–5].

Experimental studies have confirmed that in a rat model of CIH simulating OSA, typical cardiac inflammation and dysfunction are observed, characterized by significantly reduced left ventricular ejection fraction (LVEF) and left ventricular fractional shortening (LVFS), accompanied by structural dilation of the cardiac chambers [6]. This pathological process is closely associated with myocardial remodeling, in which cardiac fibrosis serves as a core element. The initiation and progression of fibrosis involve a complex network of diverse cell types and signaling pathways, such as HIF‐1*α*/ nuclear factor‐kappa B (NF‐*κ*B) and TGF‐*β* [6, 7]. At the molecular level, the NF‐*κ*B signaling pathway has been identified as a key mediator of CIH‐induced cardiovascular injury. A mechanistic review on cardiovascular disease in OSA emphasizes that this pathway coordinates the recruitment of inflammatory cells and the expression of proinflammatory mediators, thereby exacerbating myocardial injury [8]. This view is strongly supported at the cellular level; further research reveals that specific activation of NF‐*κ*B in cardiac fibroblasts under pressure overload promotes the recruitment of proinflammatory Ly6C^hi^ monocytes to the heart, directly driving the processes of myocardial inflammation and pathological remodeling [9].

The study by Adamopoulos et al. provides direct molecular evidence supporting this notion, demonstrating that advanced glycation end products activate the ERK1/2–NF‐*κ*B signaling pathway, thereby inducing the binding of the transcription factor NF‐*κ*B to the promoter region of the lysyl oxidase (LOX) gene and directly driving its transcriptional upregulation in human aortic endothelial cells [10]. LOX serves as a core regulatory enzyme in maintaining extracellular matrix homeostasis and driving pathological cardiac fibrosis [11, 12]. Its overexpression promotes irreversible covalent cross‐linking of Types I and III collagen fibers, significantly increasing myocardial stiffness, impairing ventricular compliance, and ultimately leading to both diastolic and systolic dysfunction [13]. Research indicates that CIH may mediate myocardial pathology via activation of the “NF‐*κ*B/LOX” signaling pathway: In vivo experiments have confirmed that CIH activates NF‐*κ*B in cardiovascular tissues [14], and rat models have observed cardiac inflammation, apoptosis, fibrosis, and progressive cardiac dysfunction concomitant with NF‐*κ*B upregulation [6]. This suggests that LOX activation downstream of NF‐*κ*B is a critical component of this process. Furthermore, hypoxic stress itself acts as a potent stimulus that significantly upregulates the expression of LOX and lysyl oxidase‐like 2 (LOXL2), thereby accelerating extracellular matrix remodeling and promoting the progression of pathological cardiac remodeling [15, 16].

Despite this established role of the NF‐*κ*B signaling pathway, its precise regulation under the combined hypoxic and inflammatory stress of CIH remains elusive, and targeted therapeutic strategies are lacking. Buyang Huanwu decoction (BYHW), a traditional Chinese herbal formula first recorded in Correction of Errors in Medical Classics during the Qing Dynasty, consists of seven herbs: *Astragalus membranaceus*, *Angelica sinensis*, Paeoniae Radix Rubra, Chuanxiong Rhizoma, Persicae Semen, Carthami Flos, and Pheretima. It is traditionally used to invigorate qi, activate blood circulation, and unblock meridians and has been widely applied in the treatment of poststroke sequelae, coronary heart disease, and heart failure characterized by “qi deficiency and blood stasis” [17–19].

Modern pharmacological studies have confirmed that BYHW possesses anti‐inflammatory, antioxidant, and antifibrotic properties [20, 21] and demonstrates cardioprotective effects in various models, including ischemic cardiomyopathy and pressure overload–induced heart failure [22–24]. Furthermore, mechanistic investigations have revealed that its therapeutic efficacy is closely associated with the effective suppression of the TLR4/NF‐*κ*B/NLRP3 inflammatory signaling pathway, thereby ameliorating ventricular remodeling and mitigating myocardial inflammation and fibrosis [25].

Given the pivotal role of the NF‐*κ*B/LOX signaling pathway in CIH‐induced inflammation and myocardial fibrosis, along with documented evidence that BYHW possesses anti‐inflammatory and antifibrotic properties and inhibits NF‐*κ*B pathway activation, we hypothesize that this herbal formulation may exert protective effects by modulating this pathway. However, it remains unclear whether BYHW can attenuate CIH‐induced cardiac remodeling and dysfunction through regulation of the NF‐*κ*B/LOX signaling pathway.

Therefore, this study is aimed at investigating the protective effects of BYHW against CIH‐induced cardiac dysfunction and fibrosis, as well as to explore the underlying mechanisms using a rat model of OSA‐related CIH. To evaluate BYHW′s regulatory effects on the NF‐*κ*B/LOX signaling pathway, the NF‐*κ*B agonist lipopolysaccharide (LPS) and inhibitor pyrrolidinedithiocarbamate (PDTC) were employed in combination. Cardiac function was assessed by echocardiography. Qualitative and quantitative analyses of myocardial histopathological changes were performed, with quantitative measurements including inflammatory cell count, inflammatory cell density, collagen volume fraction (CVF), and positive tissue pixel area. The protein expression and gene levels of NF‐*κ*B, LOX, and fibrosis‐related proteins (Collagens I and III) were also detected. This study is expected to provide initial insights into the anti‐inflammatory and antifibrotic mechanisms of BYHW under CIH conditions and to supply experimental evidence supporting its potential clinical application in the treatment of OSA‐associated heart failure.

## 2. Materials and Methods

### 2.1. Experimental Animals

A total of 36 male Sprague–Dawley (SD) rats, aged 10 weeks and weighing 200–250 g, were provided by the Institute of Veterinary Drug Control, China (Animal Production License No. SYXK (Beijing) 2019‐0011). The rats were housed individually in the Shenzhen Peking University–Hong Kong University of Science and Technology Medical Center under controlled conditions: temperature 20°C–26°C, relative humidity 45%–55%, good ventilation, and a 12‐h light/dark cycle. Animals had free access to standard chow and water. All procedures were approved by the Animal Welfare and Ethics Committee of Shenzhen Peking University–Hong Kong University of Science and Technology Medical Center (Approval No. 2022‐379)

### 2.2. Preparation of BYHW

BYHW was prepared according to classical composition and validated experimental protocols. The prescription consisted of *Astragalus membranaceus* var. mongholicus, *Angelica sinensis*, *Paeonia lactiflora*, *Ligusticum chuanxiong*, *Prunus persica*, *Carthamus tinctorius*, and *Pheretima aspergillum* at a crude herb ratio of 120:10:10:10:10:10:4.5 (*w*/*w*), in line with previous studies [26]. All herbs were sourced from Kangmei Pharmaceutical Co., Ltd. (Guangdong, China) and authenticated by a pharmacognosy specialist. The crude materials were coarsely ground, soaked in eight volumes of distilled water for 30 min, and then decocted for 30 min. This process was repeated with five volumes of water for a second decoction. The two filtrates were combined, passed through four layers of gauze, and concentrated under reduced pressure at temperatures below 60°C to a final concentration of 0.5265 g/mL crude drug. The decoction was stored at 4°C and freshly prepared every 7 days throughout the 5‐week intervention period.

### 2.3. Reagents and Instruments

#### 2.3.1. Reagents for Protein Extraction and Western Blotting

Protein extraction was performed using RIPA lysis buffer supplemented with a protease inhibitor cocktail (both from Addison, China). Protein concentration was determined with a bicinchoninic acid (BCA) assay kit (Addison, China). For Western blotting, the following key reagents were used: 5× loading buffer (Addison, China); Rainbow 180 broad‐spectrum protein molecular weight marker (11–180 kDa; Thermo Fisher, United States, #26617); 5× MOPS/MES running buffer and 10× transfer buffer (both from Addison, China); methanol (China National Pharmaceutical Group, China, #80080418); polyvinylidene fluoride (PVDF) membranes with pore sizes of 0.45 *μ*m (Millipore, United States, #IPVH00010) and 0.2 *μ*m (Millipore, United States, #ISEQ00010); 10× TBST (Addison, China); and blocking solution prepared with skim milk (Beyotime, China, #P0216‐1500 g). Protein bands were visualized using ECL Plus ultrasensitive chemiluminescent substrate (Biosharp, China, #BL520B).

The primary antibodies and their dilutions were as follows: mouse monoclonal anti‐Collagen I (Proteintech, United States, #66761‐1‐Ig, 1:1000); mouse monoclonal anti‐Collagen III (Proteintech, United States, #68320‐1‐Ig, 1:1000); rabbit polyclonal anti‐LOX (Boster, China, #BM5132, 1:500); rabbit monoclonal anti‐p65 (Proteintech, United States, #80979‐1‐RR, 1:1000); and mouse monoclonal anti‐*β*‐Actin (Proteintech, United States, #66009‐1‐Ig, 1:2000). Subsequently, membranes were incubated with the corresponding horseradish peroxidase (HRP)–conjugated secondary antibodies: goat anti‐rabbit IgG (Proteintech, United States, #RGAR001, 1:4000) or goat anti‐mouse IgG (Proteintech, United States, #RGAM001, 1:4000).

#### 2.3.2. Reagents for RNA Extraction and Quantitative PCR

Total RNA was extracted using TRNzol Universal Reagent (TianGen, DP424), followed by reverse transcription with the RevertAid First Strand cDNA Synthesis Kit (Thermo Scientific, K1622). Quantitative PCR was performed using 2× SYBR Green qPCR Master Mix (Selleck, B21203), and RNase/DNase‐free water (TianGen, RT121‐02) was used throughout. Additional molecular reagents included chloroform (Sinopharm, 10006818), isopropanol (Sinopharm, 80109218), anhydrous ethanol (Sinopharm, 10009218), agarose (BioWest, 111935), DNA Marker (Trans DNA Marker II, BM411), and gene‐specific primers (synthesized by Genewiz).

#### 2.3.3. Inhibitors and Stimulators for NF‐*κ*B Signaling Pathway Modulation

To modulate the NF‐*κ*B signaling pathway in vivo, the NF‐*κ*B inhibitor PDTC (Shanghai Jizhi Biochemical Technology Co., Ltd., S3633) and the classical NF‐*κ*B activator (LPS; Shanghai Jizhi Biochemical Technology Co., Ltd., AC12037) were administered via intraperitoneal injection. Previous studies have demonstrated that PDTC effectively inhibits NF‐*κ*B activation and downstream inflammatory responses, especially in LPS‐induced inflammation models [27, 28].

#### 2.3.4. Instruments and Equipment

The key instruments and equipment used in this study included:

• **Animal-related systems**: CIH exposure system (Ox‐100‐XL, Shanghai Tawain Intelligent Technology Co., Ltd., China), animal ultrasound imaging system (Vevo3100, FUJIFILM VisualSonics, Canada), and noninvasive blood pressure monitoring system (NIBP Controller, ML125, ADInstruments Pty Ltd, Australia).
• **Molecular biology instruments**: Real‐time PCR system (ABI7500), PCR thermal cycler (Veriti), microplate reader (SpectraMax M5), electrophoresis system (POWER/PAC300), and semi‐dry transfer system (Trans‐Blot SD)—all from major manufacturers based in the United States (Applied Biosystems, Molecular Devices, Bio‐RAD).
• **Histology equipment**: Tissue dehydration machine (JT‐12K) and embedding station (JB‐P5) from Wuhan Junjie Electronics Co., Ltd., China; microtome (RM2016, Leica, Shanghai, China); panoramic digital slide scanner (PANNORAMIC DESK/MIDI/250/1000, 3DHISTECH, Hungary).
• **Microscopy systems**: Optical microscope (CX‐21) and inverted microscope (IX51) from Olympus, Japan.

### 2.4. Experimental Grouping and Treatments

As this was an exploratory study, a formal a priori sample size calculation was not conducted. The group size was set at *n* = 6 per group, which aligns with established standards in our laboratory and common practice in the field for comparable pilot phenotype characterization studies. This approach adheres to the ethical principle of reduction while ensuring the generation of robust data for preliminary analysis.

Following a 1‐week acclimation period, 36 rats were randomly assigned to groups using a random number table. Specifically, each animal was assigned a unique random number, and the order of group assignment was determined by sorting these numbers in ascending order. The rats were thereby divided into six groups (*n* = 6 per group) as follows: (1) normal control (NC); (2) CIH; (3) BYHW; (4) BYHW combined with NF‐*κ*B activator (BYHW + LPS); (5) BYHW combined with NF‐*κ*B inhibitor (BYHW + PDTC); and (6) LPS alone (LPS).

Rats in Groups 2–6 were exposed to CIH using an automated hypoxic animal exposure system (Model Ox‐100‐XL, Shanghai Tawain Intelligent Technology Co., Ltd., China). The system maintained a 1‐min hypoxia–reoxygenation cycle: Nitrogen was infused for 40 s to reduce oxygen concentration to 5%–7% for 10 s, followed by oxygen supplementation for 10 s to restore ambient levels to 20.9%. CIH exposure lasted 8 h per day (from 9:00 a.m. to 5:00 p.m.) for 5 consecutive weeks. The NC group was maintained under normoxic conditions (21% oxygen) throughout the study without CIH exposure.

The dosage of 6.25 g/kg body weight per administration (equivalent to a total daily dose of 12.5 g/kg) was selected based on its established efficacy in the aforementioned study which employed an identical formulation [26]. The decoction was administered to animals by oral gavage at a volume of 20 mL/kg, twice daily. LPS, a classical NF‐*κ*B signaling pathway activator, was administered intraperitoneally at 0.1 mg/kg once daily [29]. PDTC, a selective NF‐*κ*B inhibitor, was administered intraperitoneally at 100 mg/kg once daily for 5 weeks [30]. In combination treatment groups, BYHW was administered 30 min prior to LPS or PDTC injection to ensure pharmacological interaction.

Throughout the experimental period, animals were monitored daily for general health status, including physical appearance, food and water consumption, activity, excretion patterns, body weight, and signs of respiratory distress. The order of daily treatments was not randomized; instead, a fixed sequence (descending animal ID) was implemented. Furthermore, to minimize positional confounders, all cages were systematically rotated to new positions within the housing rack each week.

### 2.5. Inclusion and Exclusion Criteria

No predefined criteria were established for the inclusion or exclusion of rats or data points in this experiment. Animal welfare was monitored throughout the study (as detailed in the Animal Anesthesia and Euthanasia section below), and no rat met the predefined humane endpoints requiring euthanasia. Therefore, all rats that completed the experimental protocol were included in the final analysis, and none were excluded. The sample size (*n*) for each group is explicitly stated in the respective figure legends.

### 2.6. Blinding

Blinding was not possible during treatment administration due to visible differences between solutions. From data collection onward, strict blinding was implemented: All in vivo assessments and tissue harvesting were performed by personnel unaware of group assignments. Subsequently, data analysis (including image processing, molecular assays, and statistics) was conducted by researchers blinded to sample identity.

### 2.7. Animal Anesthesia and Euthanasia

Rat health and welfare were monitored through daily clinical observation and weekly body weight measurements. Given that this study did not involve tumor models, the predefined humane endpoints focused on general distress indicators, including sustained weight loss exceeding 20% of baseline, severe lethargy or prostration that prevented access to food and water, and signs of severe or persistent pain such as vocalization or self‐mutilation. Any rat meeting one or more of these criteria would have been euthanized immediately. No animal met any endpoint criteria during the study, and all survived until the scheduled experimental conclusion.

All procedures involving animals were conducted in accordance with the protocol approved by the Animal Welfare and Ethics Committee of Shenzhen Peking University–Hong Kong University of Science and Technology Medical Center, as previously mentioned under Ethical Approval Number 2022‐379. Following the 5 weeks intervention period and prior to any in‐vivo measurements, rats were fasted for 2 h with free access to water. Anesthesia was administered using 3% pentobarbital sodium (40 mg/kg, intraperitoneal injection), and core body temperature was maintained between 36.5°C and 37.5°C using a temperature‐controlled heating pad. Upon loss of the toe‐pinch reflex, rats underwent subsequent in vivo procedures, including measurements by tail‐cuff plethysmography (for blood pressure and heart rate (HR)) and echocardiographic assessment. During all in vivo procedures, rats′ body temperature was maintained at 36.5°C–37°C using the heating pad, and lubricating ophthalmic ointment was applied to prevent corneal drying. Upon completion of all in vivo procedures, rats were euthanized by exsanguination via the carotid artery. Death was confirmed by the absence of heartbeat and corneal reflex, followed by a 3–5‐minute observation period prior to cardiac tissue collection.

### 2.8. Blood Pressure and HR Measurement [31]

Blood pressure and HR were measured using a noninvasive blood pressure monitoring system (NIBP Controller, ML125, ADInstruments Pty Ltd, Australia) with a tail cuff. Rats were under deep anesthesia, with core body temperature maintained at 36.5°C–37°C using the heating pad. Rats were placed in a supine or lateral position for measurements, ensuring the tail cuff was properly applied to the root of the tail. Each measurement was taken three times per rat, with each measurement lasting approximately 2–3 min. The system was set to automatic measurement mode, recording systolic blood pressure (SBP) and HR during each cycle. The mean of three measurements per rat was used for statistical analysis.

A brief rest period of 3–5 min was allowed between measurements to ensure stability, and the measurement process was conducted by a single operator blinded to group allocation.

### 2.9. Left Ventricular Structure and Function Assessment

Transthoracic echocardiography was performed using a high‐resolution imaging system (Vevo 3100, FUJIFILM VisualSonics, Toronto, Canada) equipped with a 30–40 MHz linear‐array transducer. Parasternal long‐axis views were acquired to evaluate key cardiac function parameters, including left ventricular internal dimension at end‐diastole (LVDd), left ventricular internal dimension at end‐systole (LVDs), LVEF, and LVFS. Each measurement was conducted in triplicate by an experienced operator blinded to group allocation. The mean value was used for statistical analysis.

Throughout the procedure, rats were under deep anesthesia, with core body temperature maintained at 36.5°C–37°C using the heating pad. This protocol followed established consensus guidelines for standardizing echocardiographic assessment in adult rodents [32, 33]. The Vevo 3100 system provides superior spatial and temporal resolution, making it well suited for the quantitative evaluation of ventricular geometry and contractility in preclinical models of heart failure and cardiotoxicity [34]. Additionally, the choice and dosage of anesthesia were carefully controlled to minimize their known influence on HR and myocardial contractility, in accordance with best practice recommendations [35].

### 2.10. Histological Examination of Left Ventricular Myocardium

After death was confirmed, rats were placed in a supine position and secured to the surgical board. The thoracic skin was disinfected with iodine. A midline incision was made from the abdomen to the xiphoid process using surgical scissors. The sternum was then lifted and cut bilaterally along the costal margins to open the thoracic cavity. The heart and lungs were gently removed en bloc by applying traction to the trachea with hemostatic forceps. The heart was separated from the lung tissue, and the attached great vessels and pericardial adipose tissue were carefully trimmed. Finally, the heart was rinsed with physiological saline and immediately immersed in 4% paraformaldehyde for fixation.

After fixation in 4% paraformaldehyde, myocardial tissue samples were embedded in paraffin using the JB‐P5 tissue embedding machine (Wuhan Junjie Electronics Co., Ltd.) and sectioned at 4‐*μ*m thickness with an RM2016 microtome (Leica Microsystems, Shanghai). Four sections per sample were randomly selected for hematoxylin and eosin (H&E) staining and Sirius Red staining. HE staining was performed to assess general myocardial morphology, employing hematoxylin for nuclear staining and eosin for cytoplasmic components. Sirius Red staining was conducted using a picric acid–Sirius Red solution to specifically visualize collagen fibers under polarized light microscopy, enabling assessment of myocardial fibrosis. Stained sections were examined with an Olympus CX‐21 light microscope (Olympus, Japan). These procedures followed well‐established cardiac histology protocols [36–38].

### 2.11. HE Staining Inflammatory Cell Count Quantification [39]

#### 2.11.1. Inflammatory Cell Count (Number)

Five random fields from each sample were selected for observation under a light microscope at 400x magnification, and the number of inflammatory cells in each field was counted. The types of inflammatory cells, such as neutrophils, macrophages, and lymphocytes, were recorded and counted.

#### 2.11.2. Inflammatory Cell Density

The inflammatory cell density in each field was calculated by determining the ratio of the number of inflammatory cells to the area of the field. This metric reflects the density of inflammatory cells in the tissue and helps normalize for differences in field size.

### 2.12. Sirius Red Staining CVF Quantification [39]

#### 2.12.1. Positive Area Ratio

The AI‐based digital pathology image analysis software (Aipathwell) was used to analyze each tissue section at 200x magnification. The software automatically identifies the positively stained areas and calculates the ratio of this area to the total tissue area, reflecting the extent of fibrosis.

#### 2.12.2. Positive Tissue Pixel Area

The AI software analyzes the Sirius Red stained sections and calculates the actual pixel area of the positive stained tissue, which serves as a quantitative indicator of the degree of fibrosis.

### 2.13. Western Blot Analysis

Protein was extracted from rat left ventricular tissues. Briefly, approximately 100 mg of tissue was homogenized in RIPA lysis buffer. The homogenate was incubated on ice for 30 min and centrifuged at 12,000 × *g* for 10 min at 4°C. The protein concentration of the supernatant was determined using a BCA assay kit.

Proteins (80 *μ*g per lane) were denatured, separated by SDS‐PAGE, and transferred to PVDF membranes. After blocking with 5% skim milk, the membranes were incubated overnight at 4°C with primary antibodies against Collagen I (1:1000, Proteintech, #66761‐1‐Ig), Collagen III (1:1000, Proteintech, #68320‐1‐Ig), LOX (1:500, Boster, #BM5132), p65 (NF‐*κ*B p65; 1:1000, Proteintech, #80979‐1‐RR), and *β*‐actin (1:2000, Proteintech, #66009‐1‐Ig). After washing, membranes were incubated with HRP‐conjugated secondary antibodies (1:4000, Proteintech) for 1 h at room temperature. Protein bands were visualized with ECL substrate and quantified using GelPro32 software [40, 41].

### 2.14. Real‐Time PCR Analysis

Total RNA was extracted from left ventricular tissue using TRNzol reagent (Tiangen Biotech, Beijing, China). RNA quality was confirmed by agarose gel electrophoresis and spectrophotometric analysis. Reverse transcription was performed with the RevertAid First Strand cDNA Synthesis Kit (Thermo Fisher Scientific, United States). Quantitative real‐time PCR was conducted on an ABI 7500 Real‐Time PCR System (Applied Biosystems, United States) using 2× SYBR Green qPCR Master Mix (Selleck Chemicals, United States).

Gene‐specific for NF‐*κ*B p65, LOX, Collagen I, and Collagen III were used, with *β*‐actin as an internal control. Ct values were obtained using ABI 7500 software. The *Δ*Ct was calculated by subtracting the Ct of *β*‐actin from that of the target gene. *ΔΔ*Ct was then determined by comparing the *Δ*Ct of the experimental group with the mean *Δ*Ct of the control group. Relative gene expression levels were finally calculated using the 2^–*ΔΔ*Ct^ method [42, 43] (Table 1).

**Table 1:** Primer sequences used for RT‐qPCR.

**Gene**	**Primer direction**	**Sequence (5** ^′^ **–3** ^′^ **)**	**Product size (bp)**	**Tm (°C)**
NF‐*κ*B p65	Forward (F)	GAGACCTGGAGCAAGCCATTAGC	203	63.41
	Reverse (R)	AGTGTTGGGGGCACGGTTATC		62.67

LOX	Forward (F)	AAAGGTCAGTGTAAACCCCAGC	96	60.75
	Reverse (R)	TAGGCGTGATGTCCTGTGTAGC		62.10

Collagen I	Forward (F)	CGTGGAAACCTGATGTATGCTTG	170	60.18
	Reverse (R)	TCCTATGACTTCTGCGTCTGGTG		61.99

Collagen III	Forward (F)	CCTGAACTCAAGAGCGGAGAATACTG	158	63.03
	Reverse (R)	TCAGCACCAGCATCTGTCCACC		64.79

*β*‐Actin	Forward (F)	GTCGTACCACTGGCATTGTG	180	59.2
	Reverse (R)	TCTCAGCTGTGGTGGTGAAG		59.61

*Note:* The table lists the specific primer pairs used for the amplification of target genes by real‐time quantitative PCR (RT‐qPCR), including gene names, primer directions (forward and reverse), nucleotide sequences (5 ^′^–3 ^′^), expected product sizes (bp), and melting temperatures (Tm, °C). *β*‐Actin was used as the internal reference gene.

Reaction efficiency was determined from the amplification curves. Specificity of the PCR products was confirmed by melting curve analysis at the end of each amplification run.

### 2.15. Statistical Analysis

Statistical analysis was performed using SPSS Version 27.0 (IBM Corp., Armonk, NY, United States). Normality of data distribution was assessed using the Shapiro–Wilk test, and homogeneity of variances was evaluated using Levene′s test. Data conforming to a normal distribution are expressed as mean ± standard deviation (mean ± SD) and were analyzed using one‐way analysis of variance (one‐way ANOVA). For post hoc pairwise comparisons, the least significant difference (LSD) test was applied when variances were homogeneous, and Tamhane′s T2 test was used when variances were unequal. Nonnormally distributed data are presented as median with interquartile range (median (IQR)) and were compared using the Kruskal–Wallis rank‐sum test. All statistical tests were two‐tailed, and differences were considered statistically significant at *p* < 0.05. Graphs were generated using GraphPad Prism Version 10.4 (GraphPad Software Inc., San Diego, CA, United States).

### 2.16. Protocol and Data Availability

The experimental protocol, though not prospectively registered, is available from the corresponding author upon request. The data supporting the findings of this study are presented in the article. The underlying complete datasets are available from the corresponding author on reasonable request. This study was reported in accordance with the ARRIVE Guidelines 2.0.

## 3. Results

### 3.1. Measurement of Blood Pressure and HR in Rats

Compared with the NC group, rats exposed to CIH exhibited significantly increased SBP (Figure 1a) and HR (Figure 1b) (*p* < 0.05). Treatment with BYHW reduced SBP and HR, although not significantly (*p* > 0.05 vs. the CIH group). The cotreatment with BYHW and PDTC resulted in a greater reduction, while cotreatment with BYHW and LPS attenuated the beneficial effects of BYHW. Administration of LPS alone increased SBP and HR, with no significant difference compared with the CIH group (*p* > 0.05).

Figure 1Effects of Buyang Huanwu decoction on blood pressure and heart rate in chronic intermittent hypoxia–induced rat model: (a) systolic blood pressure (SBP) and (b) heart rate (HR). Data are presented as mean ± SD (*n* = 6 per group). Group abbreviations: NC, CIH, BYHW, BYHW + LPS, BYHW + PDTC, and LPS. ^#^ *p* < 0.05 vs. NC;  ^∗^ *p* > 0.05 vs. CIH; ^&^ *p* > 0.05 vs. BYHW; and ^△^ *p* > 0.05 vs. BYHW + PDTC.

**Figure:**
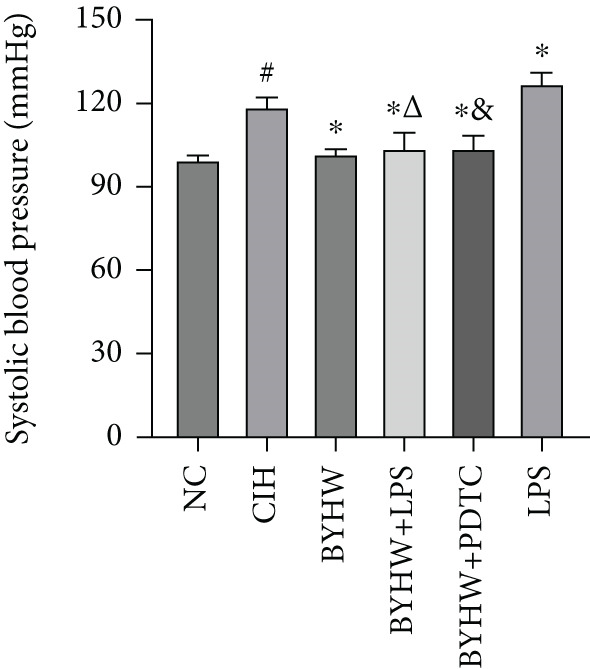
(a)

**Figure:**
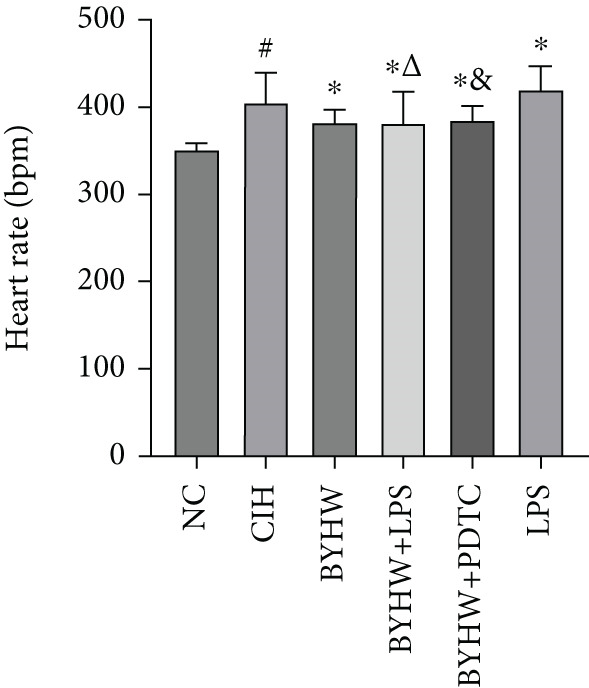
(b)

### 3.2. Echocardiographic Assessment of Cardiac Function and Structure: LVEF, LVFS, LVDd, and LVDs

Echocardiographic assessment (Figure 2a) demonstrated that CIH and LPS exposure significantly impaired cardiac function, as shown by decreased LVEF (Figure 2b) and LVFS (Figure 2c) and increased LVDd (Figure 2d) and LVDs (Figure 2e) (*p* < 0.05 vs. the NC group). Compared with the CIH group, BYHW administration significantly improved all these parameters (*p* < 0.05). The improvement was further enhanced by cotreatment with BYHW and PDTC (*p* < 0.05 vs. the BYHW group). Conversely, cotreatment with BYHW and LPS significantly attenuated the effects of BYHW (*p* < 0.05 vs. the BYHW group). LPS intervention alone worsened all parameters compared with the CIH group (*p* < 0.05).

Figure 2Evaluation of cardiac function by echocardiography in chronic intermittent hypoxia–induced rat model treated with Buyang Huanwu decoction. (a) Representative echocardiographic images of each group (*n* = 6). (b–e) Quantitative analysis of (b) LVEF, (c) LVFS, (d) LVDd, and (e) LVDs. Data are presented as mean ± SD (*n* = 6 per group). Group abbreviations: NC, CIH, BYHW, BYHW + LPS, BYHW + PDTC, and LPS. ^#^ *p* < 0.05 vs. NC;  ^∗^ *p* < 0.05 vs. CIH; ^&^ *p* < 0.05 vs. BYHW; and ^△^ *p* < 0.05 vs. BYHW + PDTC.

**Figure:**
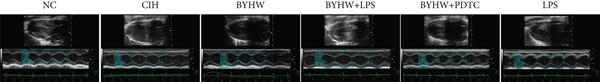
(a)

**Figure:**
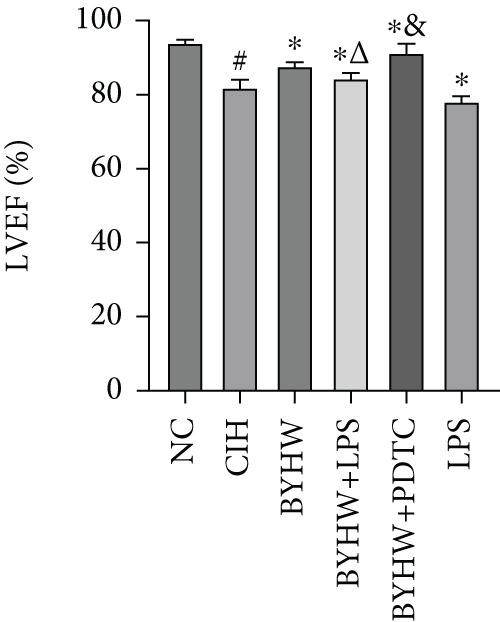
(b)

**Figure:**
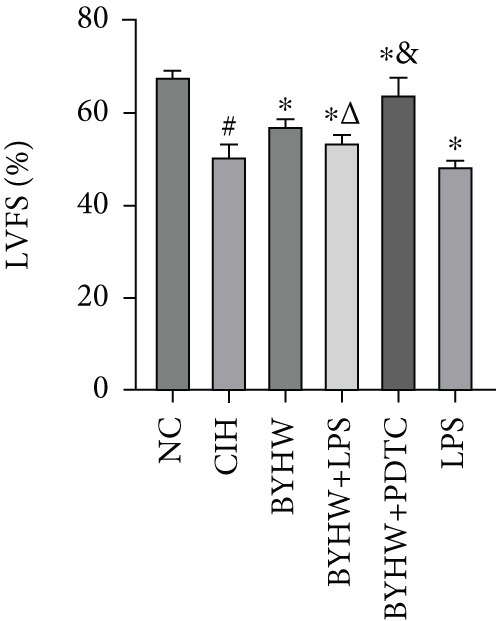
(c)

**Figure:**
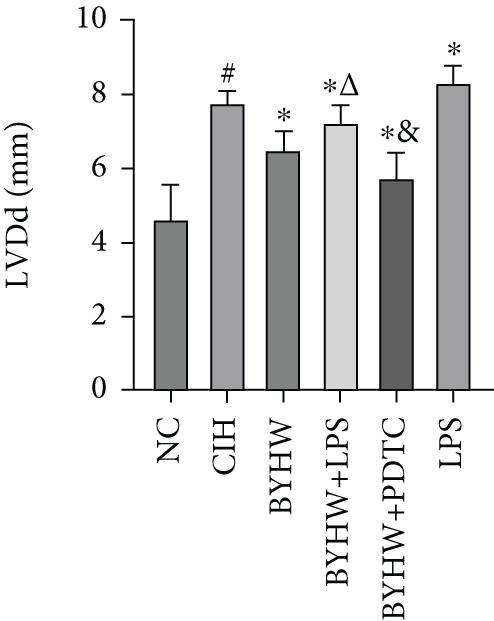
(d)

**Figure:**
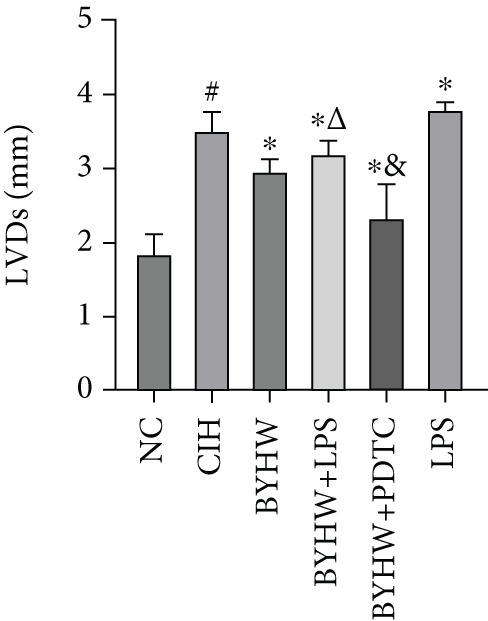
(e)

### 3.3. Histopathological Evaluation of Myocardial Tissue by H&E and Sirius Red Staining

**Figure 3:**
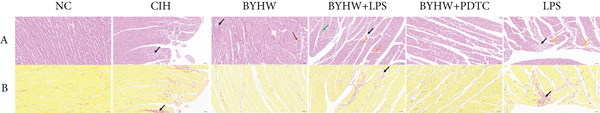
Representative histological sections of rat myocardium. (a) Hematoxylin and eosin (H&E) staining. (b) Sirius Red staining. Images were captured at 200× magnification; scale bar = 50 * μ*m. Group abbreviations: NC, CIH, BYHW, BYHW + LPS, BYHW + PDTC, and LPS. NC: myocardial tissue with typical histological architecture. CIH: connective tissue proliferation (black arrows), areas of cytoplasmic eosinophilia (green arrows), and lymphocytic infiltration. BYHW: foci of cardiomyocyte necrosis with connective tissue proliferation (black arrows) and perivascular edema (brown arrows). BYHW+LPS: cytoplasmic vacuolation (orange arrows), cytoplasmic eosinophilia (green arrows), connective tissue proliferation, and inflammatory cell infiltration. BYHW+PDTC: myocardial structure with minimal alterations and focal perivascular collagen deposition. LPS: cytoplasmic vacuolation (orange arrows) and multifocal connective tissue proliferation (black arrows).

Representative images of H&E and Sirius Red staining under low magnification (200×) are presented in Figure 3. Myocardial tissues from rats in the CIH and LPS groups exhibited evident histopathological alterations, including focal myocyte vacuolization, necrosis, and enhanced collagen deposition. In contrast, the myocardial structure was better preserved, and collagen accumulation was visibly reduced in the BYHW and BYHW + PDTC groups. The coadministration of LPS with BYHW partially attenuated these protective morphological improvements.

### 3.4. Quantification of Myocardial Inflammatory Cell Infiltration by H&E Staining

Representative high magnification (400×) H&E staining images of myocardial tissue are shown in Figure 4a. CIH exposure significantly increased inflammatory cell count (Figure 4b) and density (Figure 4c) in myocardial tissue compared with the NC group (all *p* < 0.05). Compared with the CIH group, BYHW treatment significantly reduced both inflammatory cell count and density (*p* < 0.05). The cotreatment with BYHW and PDTC resulted in a further reduction compared with the BYHW group (*p* < 0.05). Conversely, cotreatment with BYHW and LPS led to higher levels of inflammatory cell infiltration than the BYHW group (*p* < 0.05). LPS intervention alone resulted in greater inflammatory cell count and density than the CIH group (*p* < 0.05).

Figure 4Inflammatory cell infiltration in rat myocardium as revealed by H&E staining. (a) Representative high‐magnification (400× magnification; scale bar = 50 * μ*m) H&E‐stained myocardial sections. (b, c) Quantitative analysis of inflammatory cell infiltration: (b) inflammatory cell count and (c) inflammatory cell density. Data are presented as mean ± SD (*n* = 6 per group). Group abbreviations: NC, CIH, BYHW, BYHW + LPS, BYHW + PDTC, and LPS. ^#^ *p* < 0.05 vs. NC;  ^∗^ *p* < 0.05 vs. CIH; ^&^ *p* < 0.05 vs. BYHW; and ^△^ *p* < 0.05 vs. BYHW + PDTC.

**Figure:**
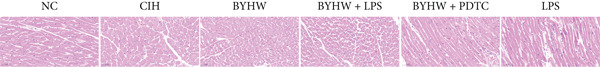
(a)

**Figure:**
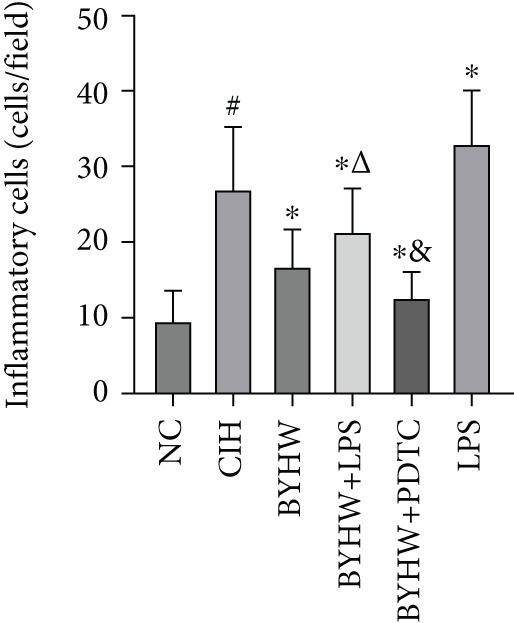
(b)

**Figure:**
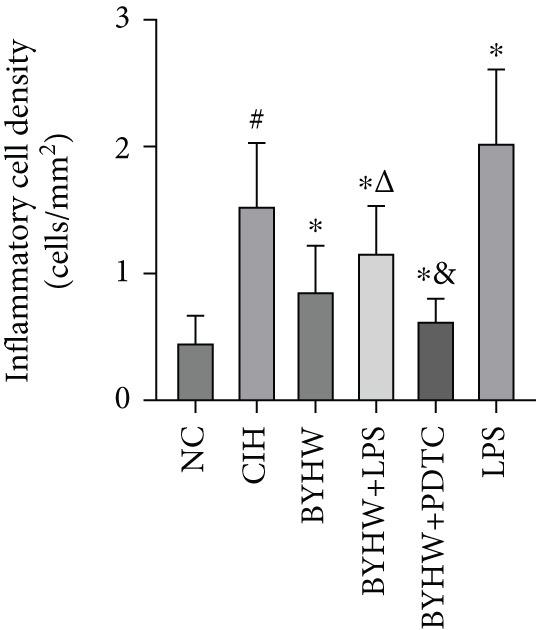
(c)

### 3.5. Quantification of Myocardial Fibrosis by Sirius Red Staining

Representative Sirius Red staining images of myocardial tissue at 200× magnification are shown in Figure 5a. The CVF results indicated increased collagen deposition in the CIH and LPS groups compared with the NC group, supported by significant elevations in the CVF‐positive area ratio (Figure 5b) and positive tissue pixel area (Figure 5c) (all *p* < 0.05). BYHW administration significantly reduced the CVF‐positive area ratio (*p* < 0.05) and positive tissue pixel area (*p* < 0.05) compared with the CIH group. The cotreatment with BYHW and PDTC resulted in a further decrease in both parameters compared with the BYHW group (*p* < 0.05). In contrast, the cotreatment with BYHW and LPS led to higher values of both metrics than BYHW alone (*p* < 0.05). LPS intervention alone resulted in the highest level of collagen deposition among all groups (*p* < 0.05).

Figure 5Myocardial collagen deposition in rat myocardium as assessed by Sirius Red staining. (a) Representative Sirius Red‐stained myocardial sections (200× magnification; scale bar = 50 * μ*m). (b, c) Quantitative analysis of collagen volume fraction (CVF): (b) CVF‐positive area ratio and (c) positive tissue pixel area. Data are presented as mean ± SD (*n* = 6 per group). Group abbreviations: NC, CIH, BYHW, BYHW + LPS, BYHW + PDTC, and LPS. ^#^ *p* < 0.05 vs. NC;  ^∗^ *p* < 0.05 vs. CIH; ^&^ *p* < 0.05 vs. BYHW; and ^△^ *p* < 0.05 vs. BYHW + PDTC.

**Figure:**
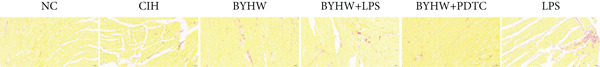
(a)

**Figure:**
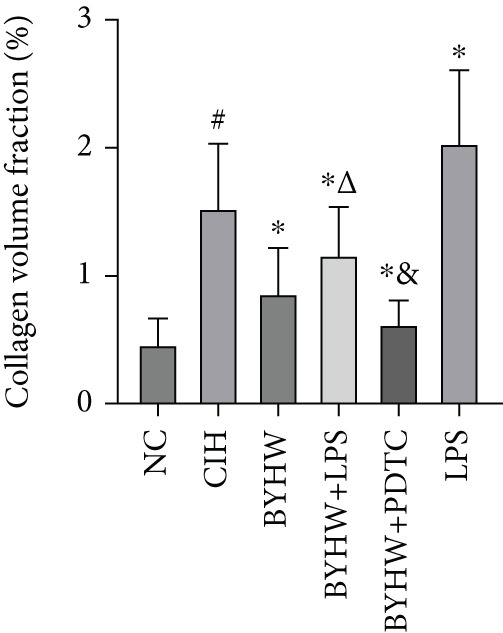
(b)

**Figure:**
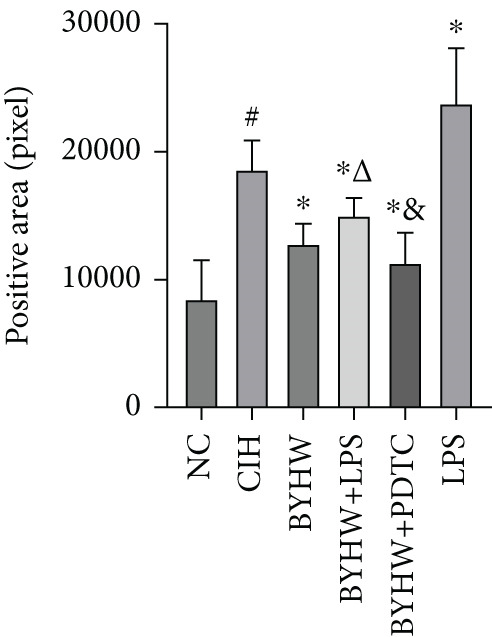
(c)

### 3.6. Quantitative Analysis of Myocardial Protein Expression by Western Blotting

Representative blot images are shown in Figure 6a, displaying the protein bands along with the internal control *β*‐actin. Western blot was used to determine the protein levels of NF‐*κ*B p65 (Figure 6b), LOX (Figure 6c), Collagen I (Figure 6d), and Collagen III (Figure 6e) in rat myocardial tissue under different conditions. Compared with the NC group, both CIH and LPS treatment significantly increased the protein levels of all four markers (*p* < 0.05). Compared with the CIH group, BYHW treatment significantly reversed the upregulation of these proteins (*P* < 0.05). The cotreatment with BYHW and PDTC further suppressed their mRNA levels compared with BYHW alone (*p* < 0.05). In contrast, cotreatment with BYHW and LPS attenuated the effects of BYHW (*p* < 0.05). LPS intervention alone significantly upregulated the levels of all four proteins compared with the CIH group (*p* < 0.05).

Figure 6Effects of Buyang Huanwu decoction on protein expression of NF‐*κ*B p65, LOX, Collagen I, and Collagen III in myocardial tissue of chronic intermittent hypoxia–induced rats. (a) Representative Western blot analysis. The blot for each target protein (NF‐*κ*B p65, LOX, Collagen I, and Collagen III) is shown directly above its corresponding *β*‐actin loading control. Samples were loaded in the order of the experimental groups (NC, CIH, BYHW, BYHW + LPS, BYHW + PDTC, and LPS). (b–e) Quantitative densitometry of protein expression normalized to *β*‐actin: (b) p65, (c) LOX, (d) Collagen I, and (e) Collagen III. Data are presented as mean ± SD (*n* = 6 per group). Group abbreviations: NC, CIH, BYHW, BYHW + LPS, BYHW + PDTC, and LPS. ^#^ *p* < 0.05 vs. NC;  ^∗^ *p* < 0.05 vs. CIH; ^&^ *p* < 0.05 vs. BYHW; and ^△^ *p* < 0.05 vs. BYHW + PDTC.

**Figure:**
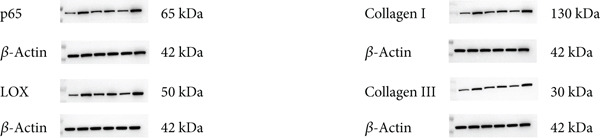
(a)

**Figure:**
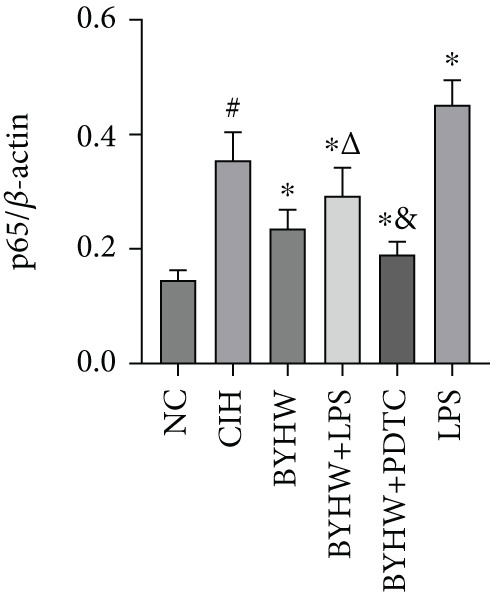
(b)

**Figure:**
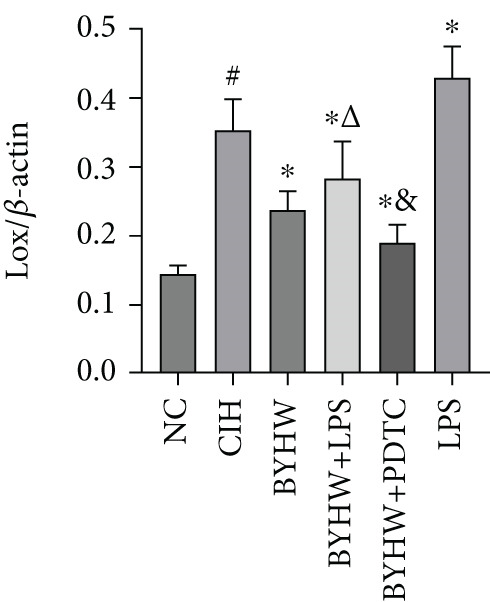
(c)

**Figure:**
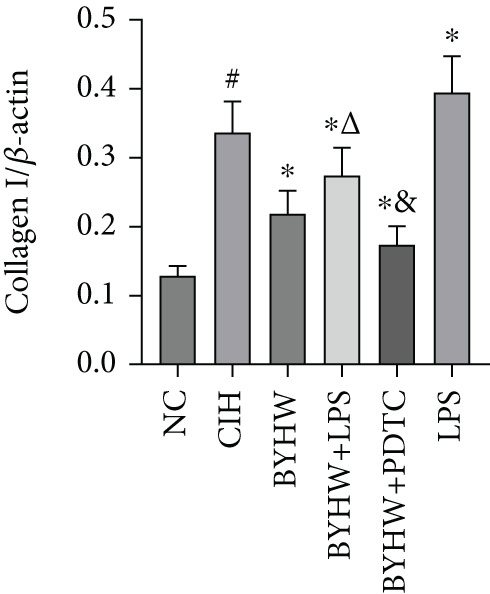
(d)

**Figure:**
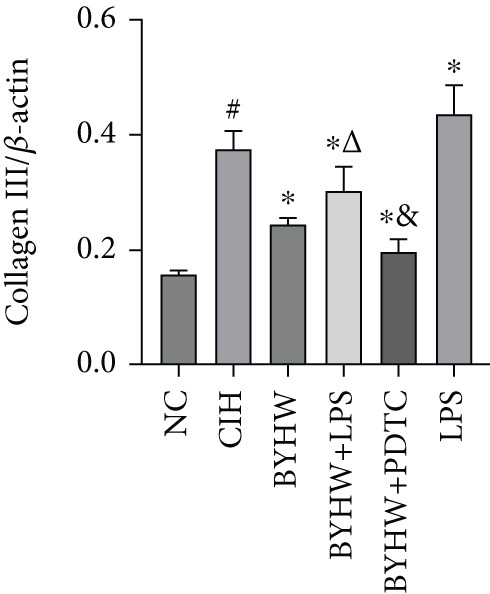
(e)

### 3.7. Quantitative mRNA Expression Profiling in Myocardial Tissue

Melting curve analysis confirmed the amplification specificity for all genes, as evidenced by the presence of single sharp peaks in all reactions (Figures 7a, 7b, 7c, 7d, and 7e). Quantitative real‐time PCR analysis revealed that compared with the NC group, the mRNA expression levels of NF‐*κ*B p65 (Figure 7f), LOX (Figure 7g), Collagen I (Figure 7h), and Collagen III (Figure 7i) were significantly elevated in the CIH group (*p* < 0.05).

Figure 7Effects of Buyang Huanwu decoction on myocardial mRNA expression of NF‐*κ*B p65, LOX, Collagen I, and Collagen III in chronic intermittent hypoxia–induced rats. (a–e) Melting curves for (a) NF‐*κ*B p65, (b) LOX, (c) Collagen I, (d) Collagen III, and (e) *β*‐actin, confirming amplification specificity. (f–i) Quantitative real‐time PCR analysis of (f) NF‐*κ*B p65, (g) LOX, (h) Collagen I, and (i) Collagen III mRNA expression levels. Data are presented as mean ± SD (*n* = 6 per group). Group abbreviations: NC, CIH, BYHW, BYHW + LPS, BYHW + PDTC, and LPS. ^#^ *p* < 0.05 vs. NC;  ^∗^ *p* < 0.05 vs. CIH; ^&^ *p* < 0.05 vs. BYHW; and ^△^ *p* < 0.05 vs. BYHW + PDTC.

**Figure:**
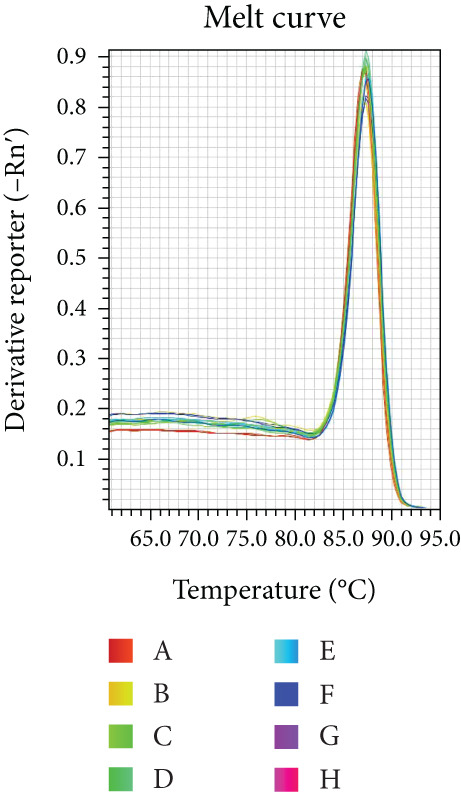
(a)

**Figure:**
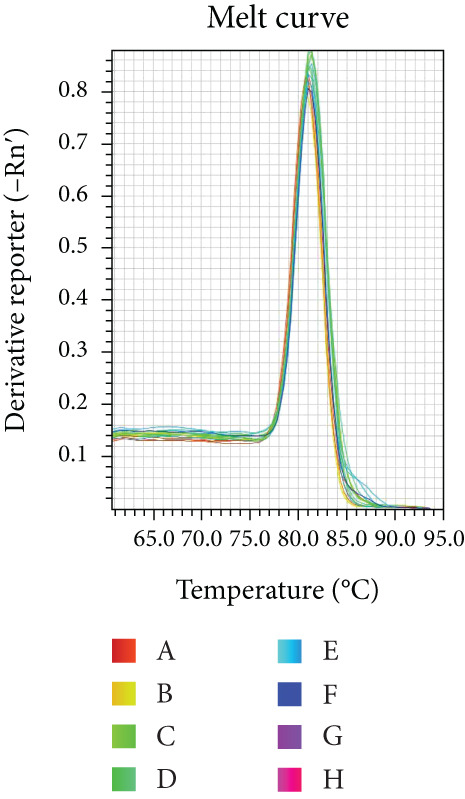
(b)

**Figure:**
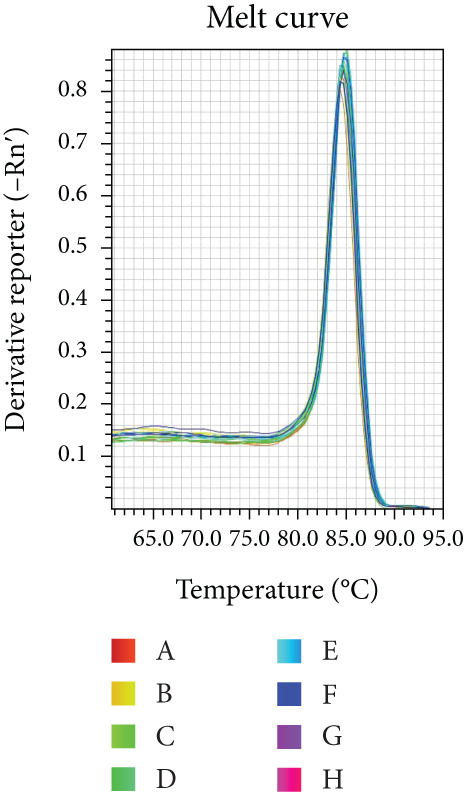
(c)

**Figure:**
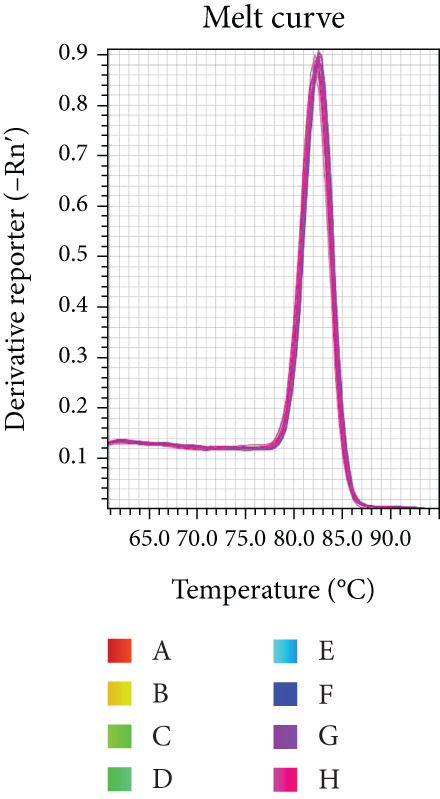
(d)

**Figure:**
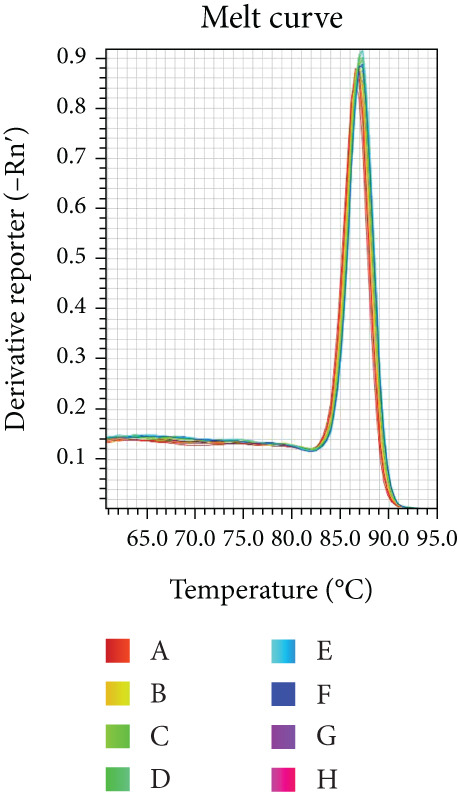
(e)

**Figure:**
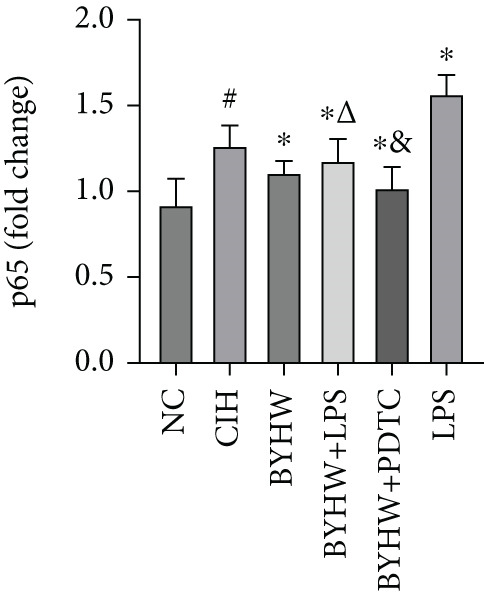
(f)

**Figure:**
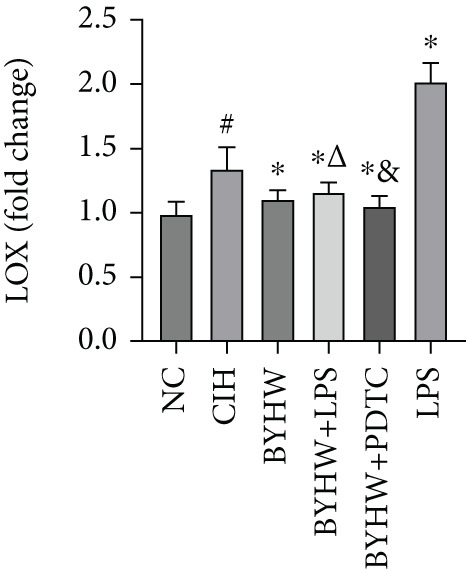
(g)

**Figure:**
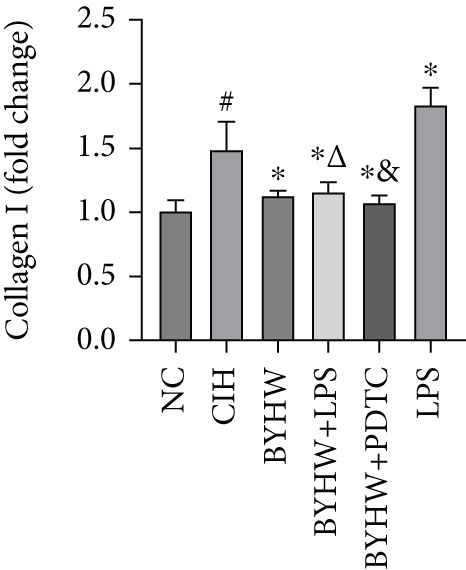
(h)

**Figure:**
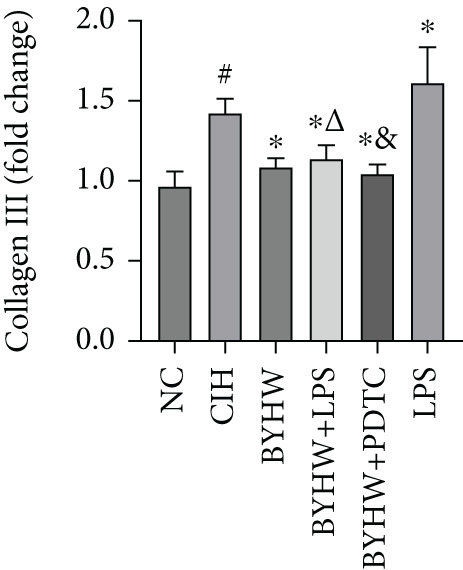
(i)

Treatment with BYHW significantly downregulated the mRNA levels of all four genes compared with the CIH group (*p* < 0.05). The cotreatment with BYHW and PDTC further suppressed their mRNA levels compared with BYHW alone (*p* < 0.05). In contrast, cotreatment with BYHW and LPS attenuated the inhibitory effect of BYHW (*p* < 0.05). LPS intervention alone significantly upregulated the mRNA levels of all four genes compared with the CIH group (*p* < 0.05).

## 4. Discussion

This study systematically evaluated the protective effects of BYHW on CIH‐induced myocardial injury and its underlying mechanisms. The results demonstrated that BYHW significantly ameliorated CIH‐induced cardiac dysfunction in rats, specifically evidenced by improved ejection fraction and fractional shortening, as well as the attenuation of left ventricular dilation. Histopathological analysis further revealed that BYHW effectively reduced inflammatory cell infiltration and collagen fiber deposition in myocardial tissue, suggesting anti‐inflammatory and antifibrotic properties. Mechanistically, BYHW treatment suppressed the expression of NF‐*κ*B, LOX, and Collagens I and III in cardiac tissue. This inhibitory effect was potentiated by coadministration with the NF‐*κ*B inhibitor PDTC but attenuated by cotreatment with the agonist LPS. Collectively, these findings indicate that the cardioprotective effect of BYHW in this CIH model is mediated, at least in part, through the suppression of the NF‐*κ*B/LOX signaling pathway.

By integrating assessments of cardiac function, histopathological analysis, and detection of molecular expression, this study established a coherent line of evidence. Based on these findings, the beneficial effects of BYHW on cardiac function and histopathology are associated with its suppression of the NF‐*κ*B/LOX signaling pathway. Specifically, BYHW treatment reduced the expression of NF‐*κ*B and LOX, as well as that of Collagens I and III, in myocardial tissue. This suppressive effect was enhanced by coadministration with PDTC and attenuated by cotreatment with LPS, strongly supporting the involvement of this pathway. This finding aligns with the previously reported anti‐inflammatory and antifibrotic properties of this formula. A prior study demonstrated that BYHW confers benefits in a myocardial infarction model by suppressing the TLR4 signaling pathway [25].

NF‐*κ*B serves as a master regulator of inflammatory responses, particularly under CIH and in the associated cardiovascular inflammation [8, 9]. Building on this study′s experimental evidence, we propose a mechanistic model wherein BYHW ameliorates CIH‐induced myocardial fibrosis by orchestrating a cascade that suppresses NF‐*κ*B, thereby downregulating LOX and ultimately reducing collagen synthesis. A pivotal component of this pathway is the direct transcriptional regulation of LOX by NF‐*κ*B, for which initial support exists in diverse biological systems. Specifically, Adamopoulos et al. provided direct evidence of NF‐*κ*B binding to the LOX gene promoter and driving its transcription in human aortic endothelial cells [10]. Furthermore, a correlative link between NF‐*κ*B and LOX expression has been documented in ovarian tissue [44]. Collectively, these independent lines of evidence from distinct organ systems strengthen the plausibility of our proposed mechanism within the CIH model.

Based on the above findings, it is hypothesized that BYHW ameliorates CIH‐induced myocardial fibrosis and cardiac dysfunction by suppressing the NF‐*κ*B/LOX signaling pathway, thereby reducing the synthesis and deposition of Collagens I and III. This is supported by the observation that coadministration of PDTC, an NF‐*κ*B inhibitor, enhanced the cardioprotective effects of BYHW, while LPS, an NF‐*κ*B agonist, attenuated them. Nevertheless, whether this pathway represents the central mechanism of BYHW′s action requires further comparative analysis and validation within a broader experimental and literature context.

Our findings position the NF‐*κ*B/LOX signaling pathway as a key target for BYHW, thereby extending and validating the established pathological mechanism wherein CIH induces cardiac injury by activating this very pathway. Previous studies have shown that intermittent hypoxia can activate NF‐*κ*B at the cellular level [45] and has been confirmed in cardiovascular tissues at the whole‐animal level [46]. Particularly in CIH rat models, the activation of NF‐*κ*B is directly related to the deterioration of cardiac function, a process that can be reversed by the NF‐*κ*B inhibitor PDTC [47]. Our study not only corroborates these previous findings but also further demonstrates that CIH‐induced cardiac remodeling occurs concurrently with the activation of the NF‐*κ*B/LOX signaling pathway.

Building upon the confirmed role of the NF‐*κ*B/LOX signaling pathway activated by CIH in injury pathogenesis, we further demonstrated that the protective effects of BYHW are consistent with those of other herbal formulations in CIH models. For instance, Shengmai San has been reported to improve CIH‐induced cardiac systolic dysfunction and structural damage in mice [48]. CIH, as a systemic pathological stimulus, affects multiple organs. Studies indicate that CIH can exacerbate allergic airway inflammation [49] and contribute to the progression of nonalcoholic fatty liver disease through mechanisms involving the activation of hypoxia‐inducible factors, among others [50]. At the metabolic level, intermittent hypoxia synergizes with abnormal glucose metabolism to promote atherosclerosis [51]. This evidence underscores the multisystemic pathological impact of CIH. Within this context, the present study, alongside the work by Song et al. demonstrating that Banxia Houpu Tang alleviates CIH‐induced cardiac ferroptotic injury [52], collectively suggests potential avenues for different herbal formulations to intervene in CIH‐induced cardiac injury.

Although interventional strategies vary, the reversal of myocardial fibrosis, a common core pathological feature in CIH‐induced cardiac injury, is likely crucial for achieving cardioprotection. In this process, LOX‐mediated collagen cross‐linking has been extensively demonstrated as a key mechanism leading to increased myocardial stiffness and diastolic dysfunction. This is corroborated by both clinical and basic research: LOX expression is upregulated in the hearts of patients with heart failure and correlates with the degree of collagen cross‐linking [53]; synchronous elevations of Type I collagen and LOX have been observed in the myocardium of patients with diastolic heart failure [54]; recent fundamental research confirms that inhibiting LOX itself effectively ameliorates pressure overload–induced cardiac dysfunction and fibrosis [55]. Collectively, these findings establish LOX as a key anticardiac fibrotic target.

Previous pharmacological research on BYHW has primarily focused on its antioxidant and anti‐apoptotic properties. Studies indicate that this formulation can activate the Keap1/Nrf2/HO‐1 signaling pathway, thereby enhancing antioxidant defenses in models such as cerebral ischemia injury and idiopathic pulmonary fibrosis [56, 57]. In CIH models, Danggui Buxue Tang has also been reported to delay vascular aging by activating the Nrf2/HO‐1 pathway [58]. Although the potential involvement of these established mechanisms is not excluded, our findings highlight the inhibition of the NF‐*κ*B/LOX signaling pathway as a critical mechanism for the anti‐inflammatory and anti‐fibrotic effects of BYHW in a CIH model. This perspective is further supported by the study of Huang et al. [59], who demonstrated that BYHW reduces oxidative stress–induced cardiomyocyte apoptosis by inhibiting 5‐lipoxygenase (5‐LOX) expression. Collectively, our findings demonstrate that the modulation of LOX family activity is a key mechanism underlying the cardioprotective effects of BYHW.

The NF‐*κ*B/LOX signaling pathway identified in our study, together with other pathways reported in the literature, reflects the multitarget pharmacological characteristics of BYHW. This is first illustrated by the distinct strategies of different formulations against CIH: for example, Danggui Buxue Tang delays vascular aging by activating the Nrf2/HO‐1 pathway [58], whereas the present study finds that BYHW protects the heart by suppressing the NF‐*κ*B/LOX signaling pathway. This suggests that antioxidant (Nrf2) and anti‐inflammatory (NF‐*κ*B) strategies may represent two primary approaches for herbal medicine interventions against CIH‐induced systemic injury. Regarding BYHW itself, its acting pathways vary depending on the disease model: In a D‐galactose–induced cardiac aging model, BYHW acts via inhibiting the CaM/CaMKII/MAPK signaling axis [60]; in a myocardial infarction model, it promotes angiogenesis through the PTEN/PI3K/Akt pathway [61]; in cerebral ischemia models, its glycoside components exert protective effects by activating the Nrf2‐mediated antioxidant pathway [62]. Furthermore, several known active components of this formulation (e.g., astragaloside IV, hydroxysafflor yellow A, and paeoniflorin) can act on pathways such as TLR4/NF‐*κ*B and JAK2/STAT1, respectively [63–65]. These results indicate that the pathways employed by BYHW are significantly regulated by the disease pathological context. Our findings identify that within the characteristic persistent inflammatory and hypoxic milieu of CIH, the NF‐*κ*B/LOX signaling pathway is markedly activated, thereby contributing to a key cardioprotective mechanism of BYHW.

In assessing the broader implications of these findings, it is important to acknowledge several limitations of the present study. First, the conclusions are derived from animal experiments, and their clinical relevance requires further validation through human studies. Second, the functional validation of the NF‐*κ*B/LOX signaling pathway relied primarily on pharmacological agents (PDTC and LPS); future investigations should employ techniques such as gene knockout or RNA interference to provide more direct genetic evidence of causality. Furthermore, the mechanistic exploration was confined to the in vivo level, and the direct regulatory effect of BYHW on this pathway has not been verified in cellular models (e.g., cardiomyocytes or cardiac fibroblasts). Integrating in vivo and in vitro evidence would provide a more comprehensive elucidation of its molecular mechanisms.

Based on the current findings and limitations, future research could pursue the following directions: First, to elucidate the mechanism, techniques such as conditional gene knockout should be employed to confirm the necessity of the NF‐*κ*B signaling pathway in BYHW′s effects. Subsequently, modern separation and analytical methods should be used to identify the specific active components that modulate this pathway. Finally, rigorous clinical studies are needed to evaluate the cardiovascular protective effects of BYHW in patients with CIH‐related disorders such as OSA.

This study provides experimental evidence that BYHW alleviated CIH‐induced myocardial injury by inhibiting the NF‐*κ*B/LOX signaling pathway, thereby providing a pharmacological basis for its modern application and suggesting potential targets for strategies against OSA‐related cardiac complications.

## 5. Conclusion

In summary, this study demonstrates that BYHW ameliorated CIH‐induced cardiac dysfunction and myocardial fibrosis in rats. The protective effects were associated with the suppression of the NF‐*κ*B/LOX signaling pathway, a finding supported by pharmacological inhibition and activation using PDTC and LPS, respectively. These results identify this signaling pathway as a mechanistic target for BYHW and provide a pharmacological foundation for further investigation into its mechanism of action in hypoxic injury.

## Disclosure

The funder had no role in the study design; data collection, analysis, and interpretation; or the decision to submit this work for publication.

## Conflicts of Interest

The authors declare no conflicts of interest.

## Author Contributions

Conceptualization: Lijun Ou. Methodology: Lijun Ou. Investigation: Lijun Ou, Chenyi Liu, Weicheng Zhao, Cairu Liu, and Ye Ning (Ye Ning played a central role in animal experiments and data collection). Formal analysis: Lijun Ou and Chenyi Liu. Writing—original draft: Lijun Ou. Writing—review and editing: Lijun Ou, Chenyi Liu, Weicheng Zhao, Cairu Liu, and Ye Ning. Supervision: Lijun Ou (as the project lead for this study). Funding acquisition: Lijun Ou (secured the primary grant).

## Funding

This work was supported by the Shenzhen Science and Technology Program, JCYJ20220531092001003.

## Data Availability

The data that support the findings of this study are available from the corresponding author upon reasonable request.
